# Quantification of Myocardial Dosimetry and Glucose Metabolism Using a 17-Segment Model of the Left Ventricle in Esophageal Cancer Patients Receiving Radiotherapy

**DOI:** 10.3389/fonc.2020.01599

**Published:** 2020-08-11

**Authors:** Xue Sha, Guanzhong Gong, Chunlei Han, Qingtao Qiu, Yong Yin

**Affiliations:** ^1^Shandong Cancer Hospital and Institute, Shandong First Medical University and Shandong Academy of Medical Sciences, Jinan, China; ^2^Turku PET Centre, Turku University Hospital, Turku, Finland

**Keywords:** myocardium, radiotherapy, ^18^F-FDG PET, 17-segment model, esophageal cancer

## Abstract

**Objective:**

Previous studies have shown that increased cardiac uptake of ^18^F-fluorodeoxyglucose (FDG) on positron emission tomography (PET) may be an indicator of myocardial injury after radiotherapy (RT). The primary objective of this study was to quantify cardiac subvolume dosimetry and ^18^F-FDG uptake on oncologic PET using a 17-segment model of the left ventricle (LV) and to identify dose limits related to changes in cardiac ^18^F-FDG uptake after RT.

**Methods:**

Twenty-four esophageal cancer (EC) patients who underwent consecutive oncologic ^18^F-FDG PET/CT scans at baseline and post-RT were enrolled in this study. The radiation dose and the ^18^F-FDG uptake were quantitatively analyzed based on a 17-segment model. The ^18^F-FDG uptake and doses to the basal, middle and apical regions, and the changes in the ^18^F-FDG uptake for different dose ranges were analyzed.

**Results:**

A heterogeneous dose distribution was observed, and the basal region received a higher median mean dose (18.36 Gy) than the middle and apical regions (5.30 and 2.21 Gy, respectively). Segments 1, 2, 3, and 4 received the highest doses, all of which were greater than 10 Gy. Three patterns were observed for the myocardial ^18^F-FDG uptake in relation to the radiation dose before and after RT: an increase (5 patients), a decrease (13 patients), and no change (6 patients). In a pairing analysis, the ^18^F-FDG uptake after RT decreased by 28.93 and 12.12% in the low-dose segments (0–10 Gy and 10–20 Gy, respectively) and increased by 7.24% in the high-dose segments (20–30 Gy).

**Conclusion:**

The RT dose varies substantially within LV segments in patients receiving thoracic EC RT. Increased ^18^F-FDG uptake in the myocardium after RT was observed for doses above 20 Gy.

## Introduction

Esophageal cancer (EC) is the 8th most common cancer and has the 6th highest cancer mortality rate worldwide (1). During the past decade, radiotherapy (RT) has become a primary treatment modality for patients with EC because of its effectiveness and relative safety. However, the heart lies near the middle esophagus and is inevitably irradiated during RT for middle-stage EC patients. Although advances in RT equipment and techniques have prolonged patient survival, delayed latent effects of radiation are currently being encountered in clinical practice (2, 3). Previous studies have reported that cardiac toxicity may even diminish the survival gains obtained from anticancer therapy (4, 5). Therefore, early observation of changes in cardiac function is extremely important for monitoring and evaluating the occurrence and development of radiation-induced heart disease (RIHD).

The heart is divided into chambers, arteries and valves, which consist of myocardial, connective, pericardial and vascular tissues. These various cardiac tissues have different radiosensitivities (6). Studies have confirmed injuries to cardiac substructures (7, 8), indicating that various dose constraints might be required (9). The myocardium is a vulnerable heart tissue, but organic injury from RT usually does not appear for several years when the symptoms of the injury have become irreversible, with no effective treatment (10). However, the interval between RT treatment and the detection of RIHD has been reported to range from months to years (11, 12). Functional imaging can monitor metabolic changes in myocardial activity before the occurrence of organic injury, and effective intervention measures can be taken at the initial stage of pathological changes (13, 14).

Currently, positron emission computed tomography (PET) imaging is considered the “gold standard” for detecting viable myocardium. Oncologic PET is usually performed in a fasting state because postprandial high blood glucose induces insulin secretion, which results in increased ^18^F-FDG uptake by muscle and fat and decreased ^18^F-FDG uptake by the tumor. In the fasting state, the ischemic myocardium can take up ^18^F-FDG, whereas normal myocardium and necrotic myocardium do not take up glucose. Under a glucose load, ^18^F-FDG is ingested by both normal and ischemic myocardium and can be used to evaluate the survival state of the myocardium (15). Although previous studies have shown that increased cardiac uptake of ^18^F-FDG on PET may be an indicator of myocardial injury after RT, only the global left ventricle (LV) was considered, and the radiation dose and ^18^F-FDG uptake in specific myocardial segments were not evaluated. Cardiac imaging studies have also increasingly found discrete focal changes in the heart. A better understanding of the effects of the radiation dose requires a more detailed assessment of the radiation dose for cardiac subvolumes.

This study has two objectives. First, the dosimetry and ^18^F-FDG uptake in oncologic PET are quantified using a 17-segment model of the LV proposed by the American Heart Association (AHA). Second, the relationship between the changes in the myocardial ^18^F-FDG uptake and the irradiated dose in EC patients who underwent radiotherapy RT is investigated (16). Our hypothesis is that the myocardial segment that receives higher doses will show increased ^18^F-FDG uptake. The confirmation of this hypothesis can be used as a preliminary basis to accurately evaluate the cardiac dose-response relationship and implement timely treatment measures in the initial stages of pathological changes.

## Materials and Methods

### Patient Selection

An institutional review board approved a retrospective review of the medical records for this analysis. Twenty-four EC patients who underwent consecutive oncologic ^18^F-FDG PET/CT scans at baseline (1–2 weeks before radiotherapy) and post-RT (2–3 months after radiotherapy) were enrolled in the study. The main inclusion criteria were as follows: (1) the heart was covered by the radiation field during the scan and (2) a fasting time >12 h prior to PET was observed. The exclusion criteria were as follows: (1) prior treatment with chemotherapy, (2) history of cardiac disease, congestive heart failure or coronary artery disease, and (3) a fasting blood glucose level higher than 150 mg/dl before the ^18^F-FDG injection.

### Radiotherapy Design

**FIGURE 1:**
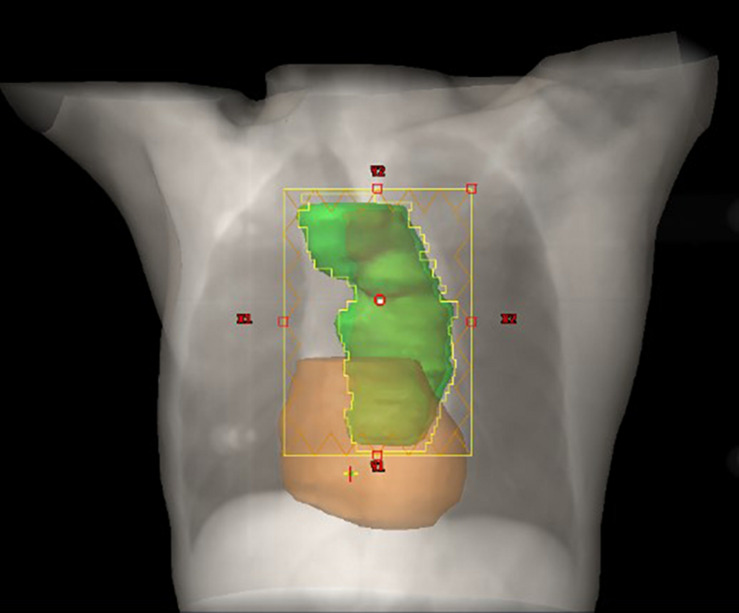
Schematic diagram of the relationship between the heart and PTV. The PTV and heart are depicted in green and color, respectively.

The prescribed dose of the planning target volume (PTV) was 60 Gy, and 95% of the PTV was required to receive the prescribed dose. The gross tumor volume (GTV) consisted of the primary tumor and metastatic regional lymph nodes observed on CT and a whole body PET/CT scan. The clinical target volume (CTV) was formed by the GTV with a 1.0-cm margin in all directions. A 0.5-cm margin for CTV was expanded to delineate the PTV. Treatment was delivered as three-dimensional conformal radiation therapy (3D-CRT) or intensity-modulated radiation therapy (IMRT) at 2.0 Gy per fraction with 6-MV photon beams. The constraints of the organ at risk (OAR) were V_20__*Gy*_ < 30% and V_30__*Gy*_ < 20% for the total lung; a maximum dose <45 Gy for the spinal cord; and V_30__*Gy*_ < 40% and V_40__*Gy*_ < 30% for the heart. Figure 1 demonstrates the PTV and radiation fields in the IMRT plan.

### ^18^F-FDG PET Image Acquisition

**FIGURE 2:**
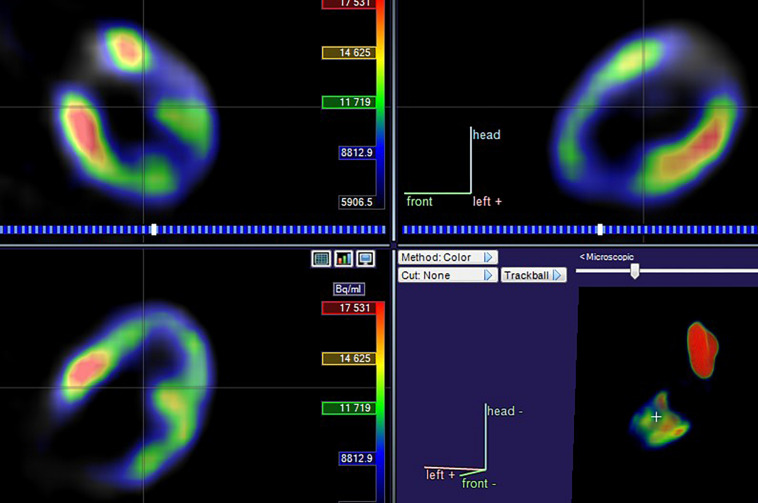
Planes of myocardial PET imaging.

The ^18^F-FDG PET/CT images were acquired from a combined PET/CT scanner (Philips Healthcare, Cleveland, OH) (Figure 2). The median fasting blood glucose level was 5.8 mmol/l (interquartile range: 5.0–6.8). Approximately 1 h after the ^18^F-FDG injection, a spiral CT was obtained, followed by a PET emission scan from the distal femur to the top of the skull. The PET emission images were corrected by the measured attenuation and reconstructed using a conventional iterative ordered-subsets expectation maximization algorithm.

### Myocardium Delineation

**FIGURE 3:**
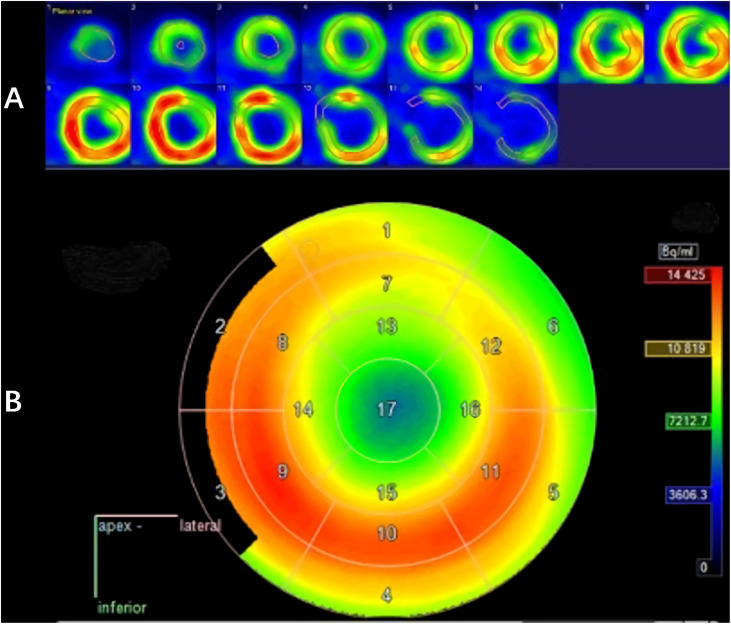
Left myocardium segmentation **(A)** and polar map **(B)**.

The LV contouring was performed by a radiologist with 20 years of experience and reviewed by two senior cardiologists. Differences in the results were resolved by consensus. Myocardium delineation was performed by Carimas software (version 2.9)^1^ based on the AHA 17-segment model, which rearranges the myocardium circumferential profile image from the apex to the base into concentric circles from the interior to the exterior, thereby projecting the entire LV myocardial onto a bull’s eye diagram. As shown in Figure 3, when contouring the LV, the myocardium was automatically divided into basal (segments 1–6), middle (segments 7–12), and apical (segments 13–16) regions.

### Dosimetry Data Acquisition

The above mentioned delineation results were used to extract the radiation dose to individual segments from the planning CT images. The individual segments were combined to determine the dose to the basal, middle and apical regions. We classified the myocardium into three groups depending on the radiation dose received: lower than 10, 10–20, and 20–30 Gy. No segment received a dose greater than 20 Gy.

### Myocardial Metabolism Measurement

The myocardial metabolism level was evaluated from the ^18^F-FDG uptake value. The 17-segment model was used to measure the myocardial metabolic parameters, which were then used in turn to carry out a quantitative analysis. For the quantitative analysis, the mean standardized uptake values (SUVs) were obtained from the baseline and the post-RT PET images. Changes in the SUV (ΔSUV) were calculated using the following formula: ΔSUV = (SUV after RT) – (SUV before RT). The SUV ratio (SUVR) was defined as the ΔSUV divided by the baseline SUV. Finally, the change in the ^18^F-FDG PET uptake was correlated with the radiation dose for the LV myocardium.

### Statistical Analysis

The SPSS 22.0 software program (SPSS, Chicago, IL, United States) was used for all the statistical tests, and the quantitative parameters were presented in terms of the mean ± standard deviation (SD). The Wilcoxon signed-rank test was used to compare the differences in different patterns of patient characteristics, and differences in ^18^F-FDG uptake values between baseline and post-RT. Only *P*-values < 0.05 were considered statistically significant.

## Results

### Patients

Between January 2016 and December 2018, 24 patients (ranging from 51 to 73 years of age, with a mean age of 61 years) with middle thoracic EC who underwent PET scans at baseline and post-RT were retrospectively enrolled in this study. Pathology reports confirmed that all the enrolled patients had esophageal squamous cell carcinoma. The heart volumes ranged from 377.1 to 805.7 with a median of 593.7 cm^3^. None of the patients showed symptomatic cardiac events during the entire planned RT process or for 3 months following RT. No significant differences in the baseline patient characteristics were observed in 3 pattern cohorts, with *P* values ranging from 0.018 to 1.000 (see Table 1).

**TABLE 1:** Patient characteristics.

Characteristic	All patients	Increased	No changes	Decreased	*P*-value
Age, years, median (range)	61 (51–73)	63 (54–67)	60 (58–63)	62 (51–73)	0.886
Sex	24	5	6	13	0.233
Male	19	3	4	12	
Female	5	2	2	1	
Tumor location					1.000
Middle	24	5	6	13	
Tumor length, cm, median(range)	6.8 (4.0–8.4)	6.9 (4.3–6.9)	6.7 (5.0–8.4)	6.8 (4.0–7.2)	0.583
Histology					1.000
Squamous cell carcinoma	24	5	6	13	
Tumor staging					0.301
I B	2	0	0	2	0.223
II	3	3	0	0	0.135
III A	4	0	0	4	0.082
IIIB	8	0	3	5	0.018
IIIC	3	0	3	0	0.135
IV	2	0	0	2	0.223
IV A	2	2	0	0	0.223
Mean heart volume, cm^3^(range)					0.067
	593.7	495.6	628.4	608.2	
	(377.1–805.7)	(470.1–495.7)	(574.5–682.3)	(377.1–805.7)	
Concurrent chemotherapy	0	0	0	0	1.000
Radiotherapy technique					0.170
IMRT	18	5	3	10	
3D-CRT	6	0	3	3	
Prescribed dose	60 Gy	60 Gy	60 Gy	60 Gy	1.000
No. of fractions	2 Gy	2 Gy	2 Gy	2 Gy	1.000

*IMRT, intensity-modulated radiation therapy; 3D-CRT, 3-dimensional conformal radiation therapy.*

### Doses to the Left Myocardial Segments

The median maximum, mean, and minimum LV irradiation doses were 18.14 ± 9.08, 6.54 ± 4.07, and 2.59 ± 2.23 Gy, respectively. Valuable data were recorded for discrete areas of the LV. Significant differences were observed in the dose distribution for the LV: the median mean and median maximum doses were both higher in the basal region (9.59 and 16.12 Gy, respectively) than in the middle region (6.54 and 11.53 Gy, respectively) and the apical region (5.09 and 13.17 Gy, respectively). These results were mirrored for the segments of the left myocardium, with the basal segments receiving the highest doses (segments 1, 2, 3, and 4). Table 2 shows the doses to the individual myocardium segments in the polar map.

**TABLE 2:** Irradiation dose and FDG uptake of individual segment in the myocardial 17-segment model.

Segment	Mean dose, Gy (range)	SUV		SUVR (%)	*P*-value
		Baseline	Post-RT		
**Basal**					
1	12.26 (1.89–24.63)	3.78 (2.13–6.81)	2.82 (1.24–4.59)	–19.14	0.217
2	12.25 (1.46–28.27)	4.29 (2.65–7.57)	3.33 (1.41–6.12)	–18.24	0.378
3	11.58 (0.62–24.54)	4.28 (2.38–7.70)	3.22 (1.12–7.38)	–21.91	0.304
4	10.07 (0.45–27.72)	3.68 (2.03–6.42)	2.85 (0.97–6.86)	–20.84	0.398
5	8.69 (0.53–23.65)	3.73 (2.10–5.91)	3.11 (1.48–8.14)	–17.29	0.292
6	7.84 (1.01–15.35)	3.38 (2.07–5.80)	2.65 (1.26–5.83)	–17.14	0.156
**Middle**					
7	7.64 (1.14–20.79)	3.40 (1.84–6.52)	2.53 (1.19–4.12)	–20.12	0.084
8	10.13 (0.96–25.88)	3.67 (1.87–7.05)	2.74 (1.26–5.01)	–19.67	0.090
9	8.48 (0.45–19.53)	4.09 (2.16–7.44)	2.97 (1.19–6.86)	–24.74	0.040
10	7.44 (0.32–19.68)	3.87 (2.16–7.39)	2.86 (1.04–7.16)	–24.76	0.045
11	5.92 (0.36–20.86)	3.73 (2.11–6.59)	2.94 (1.59–7.32)	–21.70	0.020
12	5.85 (0.64–10.89)	3.68 (2.18–6.62)	2.80 (1.22–5.31)	–22.79	0.033
**Apical**					
13	4.38 (0.61–8.33)	3.20 (1.91–5.76)	2.35 (1.11–4.53)	–24.88	0.019
14	6.16 (0.42–13.51)	3.25 (1.85–6.19)	2.39 (0.89–5.15)	–23.45	0.047
15	6.59 (0.24–20.64)	3.20 (2.00–5.80)	2.41 (1.02–5.26)	–22.59	0.024
16	4.78 (0.36–9.49)	3.11 (1.95–5.73)	2.30 (1.11–4.99)	–24.93	0.005
17	3.54 (0.49–6.57)	2.68 (1.66–5.02)	1.96 (0.86–4.13)	–24.09	0.007

### ^18^F-FDG Uptake of Left Myocardial Segments

**FIGURE 4:**
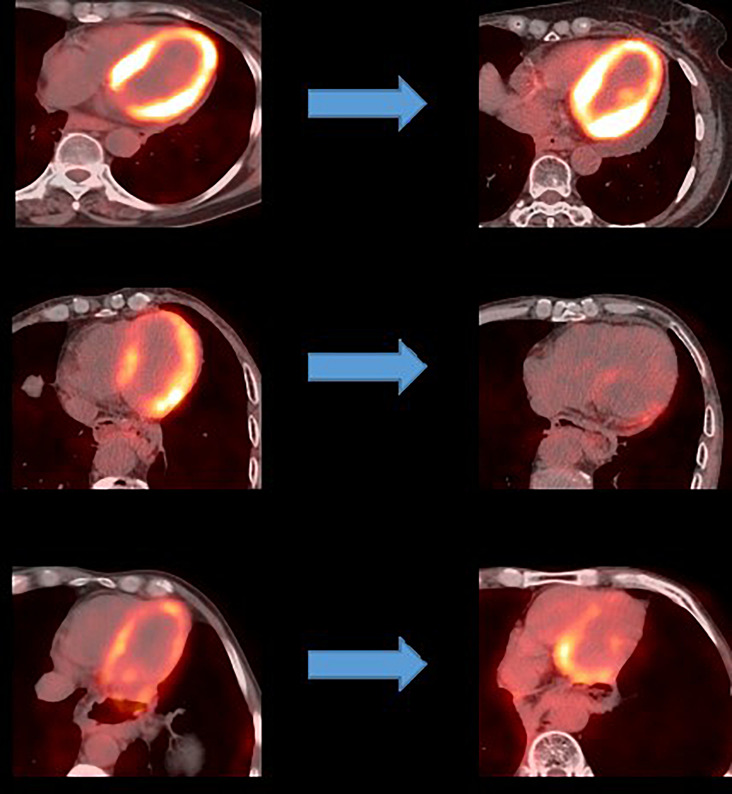
Baseline and post-RT PET imaging in patients with three myocardial accumulation patterns.

An analysis of the changes in the ^18^F-FDG uptake at two time points showed three patterns for the overall myocardial accumulation of ^18^F-FDG uptake related to the radiation dose (Figure 4). A subsequent quantitative analysis demonstrated that ^18^F-FDG uptake increased in 5 patients (average SUVR: 16.68%), decreased in 13 patients (average SUVR: −41.38%) and did not change significantly in 6 patients (average SUVR: −5.53%). However, directly focusing on specific LV segments showed that the post-RT uptake of the 17 segments tended to decrease relative to the baseline uptake: the post-RT uptake decreased in the basal, middle and apical regions by 19.09 ± 1.77, 22.30 ± 2.01, and 23.99 ± 0.89%, respectively. In agreement with these results, the ^18^F-FDG uptake of the segmental myocardium decreased significantly in segments 9–17 in the apical region (*P* < 0.05). Table 2 provides a detailed summary of baseline and post-RT ^18^F-FDG uptake for the baseline and post-RT in the left myocardium segments.

### Changes of ^18^F-FDG Uptake With Different Dose Ranges

We directly paired the radiation dose and the ^18^F-FDG uptake of the LV segments using the AHA 17-segment model. In our study, the numbers of segments that received doses in the ranges of 0–10, 10–20, and 20–30 Gy were 296, 74, and 38, respectively. ^18^F-FDG uptake in the segments receiving 0–10 and 10–20 Gy decreased by 28.93 and 12.12% after RT, respectively. The ^18^F-FDG uptake in the segments receiving 20–30 Gy increased by 7.24% after RT.

## Discussion

Subacute changes following thoracic RT have been demonstrated in discrete areas of the LV, and guidelines have been recently developed to help determine the dose to subvolumes of the LV (8, 17). Therefore, the radiation dose to cardiac subvolumes needs to be more accurately quantified to better understand the effect of the radiation dose. Previous studies have calculated cardiac doses by using modeled patients with recreated 2D treatment fields and by employing set geometric rules to define cardiac subvolumes or by using anatomical landmarks to divide the heart into standard axial imaging planes (18–20). The primary strength of the present study is that the dosimetric values and ^18^F-FDG uptake were evaluated by performing a segmental analysis and directly pairing the radiation dose and ^18^F-FDG uptake in the myocardial segments using the 17-segment AHA model.

The 17-segment model method proposed by the AHA aims to accurately divide the LV according to the anatomical structure of the heart and is the closest scheme available to that visualized by clinical ultrasound and radionuclide myocardial imaging (16). Erven et al. (21) discussed the feasibility of dividing the LV into 17 segments but did not report the doses for each segment. Tang et al. (22) used a 17-segment model to show that the dose distribution varied across LV subregions in breast cancer patients. The middle and anterior apical segments (segments 7 and 13) and the LV apical regions (segments 13, 14, 15, 16, and 17) received higher radiation doses than the other segments. The results of the present study also show a heterogeneous dose to the LV for EC patients. The basal region (segments 1, 2, 3, and 4) received a higher radiation dose than the apical and middle regions. Such reporting of specific regional dose delivery provides the most accurate spatial description of delivered radiation doses to the heart, particularly during the middle stages of EC when the LV is very frequently exposed. The use of automatic software sketching has reduced the sketching error between observers. In addition, analyzing the dose value and the ^18^F-FDG uptake of the myocardium under the same LV-VOI conditions produced a highly accurate dose-response relationship. This analytical method accurately describes the radiation doses to the myocardium and its subspaces. Further understanding of cardiotoxicity requires the precise matching of the radiation dose to regional imaging defects, which underlines the need for determining the dose distribution in detail (23).

Changes in the ^18^F-FDG uptake of the segments of the myocardium were also analyzed in the current study. To suppress the physiological myocardial accumulation of ^18^F-FDG, we selected patients who fasted for ≥18 h prior to the ^18^F-FDG PET scan, both pre- and post-RT. Ishida et al. (24) reported that the physiological accumulation of ^18^F-FDG in the irradiated myocardium was suppressed in *a* ≥ 18-h fasting group compared with *a* < 18-h fasting group. Suppression of physiological myocardial ^18^F-FDG accumulation appeared to facilitate the detection of abnormal myocardial ^18^F-FDG accumulation. Cardiotoxicity related to external RT is a recognized phenomenon in clinical practice and has traditionally been investigated by radionuclide ventriculography or gated blood pool imaging (popularly known as the multigated acquisition [MUGA] scan) and 2D echocardiography. By comparison, the observation of ^18^F-FDG PET/CT in the present study is novel and may have considerable significance if translated into clinical practice and utilized to monitor the effect of RT in a patient-specific manner (25). In this study, three radiation dose patterns were observed for the myocardial accumulation of ^18^F-FDG on PET: an increase, a decrease or no change. Although the exact molecular pathway remains to be determined, in-depth research in this field can be applied to the specific diagnosis and management of different patients such that corresponding preventive or therapeutic measures can be provided.

Last, the changes in ^18^F-FDG uptake by myocardial segments were analyzed for different dose ranges. Very few studies have explored this topic to date, and the two existing publications on RT in EC present conflicting results. Jingu et al. (26) considered focally increased ^18^F-FDG uptake in the basal myocardium after RT to be an indicator of radiation-induced cardiac damage, whereas Konski et al. (27) found no correlation between the percent change in the myocardial SUV and cardiac toxicity. A recent study by Evans et al. (25) found that in lung cancer patients treated with stereotactic body RT (SBRT), ^18^F-FDG uptake increased when the 20 Gy isodose line exceeded 5 cm^3^ of the heart. By comparison, the results of the present study show that ^18^F-FDG uptake in myocardial segments receiving a low dose (0–20 Gy) decreased after RT, whereas ^18^F-FDG uptake in the myocardial segments receiving 20–30 Gy increased after RT. Therefore, minimizing the volumes of myocardium being irradiated by more than 20 Gy can be expected to reduce the incidence of myocardial injury. However, this hypothesis needs to be evaluated in larger scale prospective studies with longer follow-up times.

The current study has several limitations. First, the 5-year incidence rate of heart disease or pericardial effusion following RT ranges from 11.1 to 13.8% (28–30). Further follow-up is required to reveal the clinical significance of abnormal myocardial accumulation of ^18^F-FDG following RT. Second, the heart is a mobile structure, and its location on 2 PET scans may vary both interfractionally and intrafractionally relative to the planned CT scan, rendering the regional dose distribution corresponding to ^18^F-FDG PET a good approximation, at best. Third, strictly enrolling patients who fasted for >18 h before undergoing PET scans resulted in a limited number of patients for this retrospective study. More patients need to be evaluated in future prospective studies.

Despite these drawbacks, we consider our data on cardiac toxicity during RT to be robust. Currently, we are not suggesting that the study results should be used to modify current treatment modalities. However, we are recommending that efforts should be made to reduce the cardiac dose and irradiated volumes during thoracic RT, which may benefit patients, especially those with favorable prognoses. Very few publications on the subject of this study are currently available. Thus, this investigation is the first of its kind: specific cardiac changes on PET/CT are related to dose information to detect myocardial activity in early stage post-RT. Although none of the investigated patients experienced symptomatic cardiac events between receiving RT and 3 months following RT, we consider a longer follow-up with higher numbers of patients to be essential for assessing the clinical significance of the considered abnormalities. We recommend that considerable effort should be expended to identify means of improving RT techniques to further minimize incidental irradiation of the heart.

## Conclusion

Radiotherapy doses vary substantially within specific LV segments in the setting of thoracic EC RT. Increased ^18^F-FDG uptake in the myocardium after RT was observed when receiving a dose higher than 20 Gy. Determining the ^18^F-FDG uptake and corresponding RT dose in the LV segments can help to guide focus in the diagnosis of radiation-induced cardiac toxicity.

## Data Availability Statement

The raw data supporting the conclusions of this article will be made available by the authors, without undue reservation.

## Ethics Statement

The retrospective study was reviewed and approved by The Ethics Committee (IRB) at Shandong Cancer Hospital and Institute. After a vote (Total 11, Agree 11, Disagree 0), the IRB agreed that the study followed the guidelines of Good Clinical Practice (GCP) and that the research could be conducted at Shandong Cancer Hospital and Institute (No. 201807013).

## Author Contributions

XS designed the study and wrote the initial draft of the manuscript. GG and CH contributed to the design, analysis and interpretation of the data, and assisted in the preparation of the manuscript. QQ and YY contributed to the data collection and interpretation, and critically reviewed the manuscript. All authors approved the final version of the manuscript and have agreed to be accountable for all aspects of the work and for ensuring that questions related to the accuracy or integrity of any part of the work are appropriately investigated and resolved.

## Conflict of Interest

The authors declare that the research was conducted in the absence of any commercial or financial relationships that could be construed as a potential conflict of interest.

## Footnotes

1 www.turkupetcentre.fi/carimas

## References

[1] Ferlay J Shin HR Bray F Forman D Mathers C Parkin DM Estimates of worldwide burden of cancer in 2008: GLOBOCAN 2008. *Int J Cancer.* (2010) 127:2893–917. 10.1002/ijc.25516 21351269

[2] Hendry JH Akahoshi M Wang LS Lipshultz SE Stewart FA Trott KR. Radiation-induced cardiovascular injury. *Radiat Environ Biophys.* (2008) 47:189–93. 10.1007/s00411-007-0155-7 18193445

[3] Darby SC Ewertz M McGale P Bennet AM Blom-Goldman U Brønnum D Risk of ischemic heart disease in women after radiotherapy for breast cancer. *N Engl J Med.* (2013) 368:987–98. 10.1056/NEJMoa1209825 23484825

[4] Curigliano G Cardinale D Dent S Criscitiello C Aseyev O Lenihan D Cardiotoxicity of anticancer treatments: Epidemiology, detection, and management. *CA Cancer J Clin.* (2016) 66:309–25. 10.3322/caac.21341 26919165

[5] Hooning MJ Aleman BM van Rosmalen AJ Kuenen MA Klijn JG van Leeuwen FE. Cause-specific mortality in long-term survivors of breast cancer: A 25-year follow-up study. *Int J Radiat Oncol Biol Phys.* (2006) 64:1081–91. 16446057 10.1016/j.ijrobp.2005.10.022

[6] Gillette EL McChesney SL Hoopes PJ. Isoeffect curves for radiation-induced cardiomyopathy in the dog. *Int J Radiat Oncol Biol Phys.* (1985) 11:2091–7. 4066441 10.1016/0360-3016(85)90089-6

[7] Taylor C McGale P Brønnum D Correa C Cutter D Duane FK Cardiac structure injury after radiotherapy for breast cancer: cross-sectional study with individual patient data. *J Clin Oncol.* (2018) 36:2288–96. 10.1200/JCO.2017.77.6351 29791285 PMC6067799

[8] Darby SC Cutter DJ Boerma M Constine LS Fajardo LF Kodama K Radiation-related heart disease: current knowledge and future prospects. *Int J of Radiat Oncol Biol Phys.* (2010) 76:656–65. 10.1016/j.ijrobp.2009.09.064 20159360 PMC3910096

[9] Gagliardi G Constine LS Moiseenko V Correa C Pierce LJ Allen AM Radiation dose–volume effects in the heart. *Int J Radiat Oncol Biol Phys.* (2010) 76:S77–85. 10.1016/j.ijrobp.2009.04.093 20171522

[10] Lally BE Detterbeck FC Geiger AM Thomas CR Machtay M Miller AA The risk of death from heart disease in patients with nonsmall cell lung cancer who receive postoperative radiotherapy: analysis of the surveillance, epidemiology, and end results database. *Cancer.* (2010) 110:911–7. 10.1002/cncr.22845 17620279

[11] Marks LB Yu X Prosnitz RG Zhou SM Hardenbergh PH Blazing M The incidence and functional consequences of RT-associated cardiac perfusion defects. *Int J Radiat Oncol Biol Phys.* (2005) 63:214–23. 10.1016/j.ijrobp.2005.01.029 16111592

[12] Sioka C Exarchopoulos T Tasiou I Tzima E Fotou N Capizzello A Myocardial perfusion imaging with (99 m) Tc-tetrofosmin SPECT in breast cancer patients that received postoperative radiotherapy: a case-control study. *Radiat Oncol.* (2011) 6:151. 10.1186/1748-717X-6-151 22067743 PMC3222615

[13] Karam HM Radwan RR. Metformin modulates cardiac endothelial dysfunction, oxidative stress and inflammation in irradiated rats: A new perspective of an antidiabetic drug. *Clin Exp Pharmacol Physiol.* (2019) 46:1–9. 10.1111/1440-1681.13148 31357226

[14] Schultz-Hector S Trott KR. Radiation-induced cardiovascular diseases: is the epidemiologic evidence compatible with the radiobiologic data? *Int J Radiat Oncol Biol Phys.* (2007) 67:10–8. 10.1016/j.ijrobp.2006.08.071 17189062

[15] Hardenbergh PH Munley MT Bentel GC Kedem R Borges-Neto S Hollis D. Cardiac perfusion changes in patients treated for breast cancer with radiation therapy and doxorubicin: preliminary results. *Int J Radiat Oncol Biol Phys.* (2001) 49:1023–8. 10.1016/s0360-3016(00)01531-5 11240243

[16] Cerqueira MD Weissman NJ Dilsizian V Jacobs AK Kaul S Laskey WK Standardized myocardial segmentation and nomenclature for tomographic imaging of the heart. A statement for healthcare professionals from the cardiac imaging committee of the council on clinical cardiology of the american heart association. *J Nucl Cardiol.* (2002) 105:539–42. 10.1067/mnc.2002.123122 11815441

[17] Luijk P Faber H Meertens H Schippers JM Langendijk JA Brandenburg S The impact of heart irradiation on dose–volume effects in the rat lung. *Int J Radiat Oncol Biol Phys.* (2007) 69:552–9. 10.1016/j.ijrobp.2007.05.065 17869668

[18] Taylor CW Nisbet A McGale P Darby SC. Cardiac exposures in breast cancer radiotherapy: 1950s–1990s. *Int J Radiat Oncol Biol Phys.* (2007) 69:1484–95. 10.1016/j.ijrobp.2007.05.034 18035211

[19] Wollschläger D Karle H Stockinger M Bartkowiak D Bührdel S Merzenich H Radiation dose distribution in functional heart regions from tangential breast cancer radiotherapy. *J Eur Soc Therap Radiol Oncol.* (2016) 119:65–70. 10.1016/j.radonc.2016.01.020 26874543

[20] Johansen S Tjessem KH Fossa K Bosse G Danielsen T Malinen E Dose distribution in the heart and cardiac chambers following 4-field radiation therapy of breast cancer: a retrospective study. *Breast Cancer..* (2013) 7:41–9. 10.4137/BCBCR.S11118 23589693 PMC3615991

[21] Erven K Florian A Slagmolen P Sweldens C Jurcut R Wildiers H Subclinical cardiotoxicity detected by strain rate imaging up to 14 months after breast radiation therapy. *Int J Radiat Oncol Biol Phys.* (2013) 85:1172–8. 10.1016/j.ijrobp.2012.09.022 23149005

[22] Tang S Otton J Holloway L Delaney PG Liney G George A Quantification of cardiac subvolume dosimetry using a 17 segment model of the left ventricle in breast cancer patients receiving tangential beam radiotherapy. *Radiother Oncol.* (2019) 132:257–65. 10.1016/j.radonc.2018.09.021 30446318

[23] Duane F Aznar MC Bartlett F Cutter DJ Darby SC Jagsi R A cardiac contouring atlas for radiotherapy. *J Eur Soc Therap Radiol Oncol.* (2017) 122:416–22. 10.1016/j.radonc.2017.01.008 28233564 PMC5356506

[24] Ishida Y Sakanaka K Itasaka S Nakamoto Y Togashi K Mizowaki T Effect of long fasting on myocardial accumulation in 18F-fluorodeoxyglucose positron emission tomography after chemoradiotherapy for esophageal carcinoma. *J Radiat Res.* (2017) 59:182–9. 10.1093/jrr/rrx076 PMC5951029 29281031

[25] Evans JD Gomez DR Chang JY Gladish GW Erasmus JJ Rebueno N Cardiac 18F-fluorodeoxyglucose uptake on positron emission tomography after thoracic stereotactic body radiation therapy. *Radiother Oncol.* (2013) 109:82–8. 24016676 10.1016/j.radonc.2013.07.021

[26] Jingu K Kaneta T Nemoto K Ichinose A Oikawa M Takai Y The utility of 18F-fluorodeoxyglucose positron emission tomography for early diagnosis of radiation-induced myocardial damage. *Int J Radiat Oncol Biol Phys.* (2006) 66:845–51. 10.1016/j.ijrobp.2006.06.007 17011456

[27] Konski A Li T Christensen M Cheng JD Yu JQ Crawford K Symptomatic cardiac toxicity is predicted by dosimetric and patient factors rather than changes in 18F-FDG PET determination of myocardial activity after chemoradiotherapy for esophageal cancer. *Radiother Oncol.* (2012) 104:72–7. 10.1016/j.radonc.2012.04.016 22682539 PMC3389132

[28] Ishikura S Nihei K Ohtsu A Boku N Hironaka S Mera K Long-term toxicity after definitive chemoradiotherapy for squamous cell carcinoma of the thoracic esophagus. *J Clin Oncol.* (2003) 21:2697–702. 10.1200/JCO.2003.03.055 12860946

[29] Beukema JC van Luijk P Widder J Langendijk JA Muijs CT. Is cardiac toxicity a relevant issue in the radiation treatment of esophageal cancer? *Radiother Oncol.* (2015) 114:85–90. 10.1016/j.radonc.2014.11.037 25554226

[30] Ito H Itasaka S Sakanaka K Araki N Mizowaki T Hiraoka M. Long-term complications of definitive chemoradiotherapy for esophageal cancer using the classical method. *J Radiat Res.* (2017) 58:106–13. 10.1093/jrr/rrw078 27475126 PMC5321186

